# Influence of spatial frequency in visual stimuli for cVEP-based BCIs: evaluation of performance and user experience

**DOI:** 10.3389/fnhum.2023.1288438

**Published:** 2023-11-10

**Authors:** Álvaro Fernández-Rodríguez, Víctor Martínez-Cagigal, Eduardo Santamaría-Vázquez, Ricardo Ron-Angevin, Roberto Hornero

**Affiliations:** ^1^UMA-BCI Group, Departamento de Tecnología Electrónica, Universidad de Málaga, Malaga, Spain; ^2^Grupo de Ingeniería Biomédica, Universidad de Valladolid, Valladolid, Spain; ^3^Centro de Investigación Biomédica en Red de Bioingeniería, Biomateriales y Nanomedicina, Valladolid, Spain

**Keywords:** brain-computer interface (BCI), code-modulated visual evoked potential (c-VEP), stimulus, spatial frequency, checkerboard, visual fatigue

## Abstract

Code-modulated visual evoked potentials (c-VEPs) are an innovative control signal utilized in brain-computer interfaces (BCIs) with promising performance. Prior studies on steady-state visual evoked potentials (SSVEPs) have indicated that the spatial frequency of checkerboard-like stimuli influences both performance and user experience. Spatial frequency refers to the dimensions of the individual squares comprising the visual stimulus, quantified in cycles (i.e., number of black-white squares pairs) per degree of visual angle. However, the specific effects of this parameter on c-VEP-based BCIs remain unexplored. Therefore, the objective of this study is to investigate the role of spatial frequency of checkerboard-like visual stimuli in a c-VEP-based BCI. Sixteen participants evaluated selection matrices with eight spatial frequencies: C001 (0 c/°, 1×1 squares), C002 (0.15 c/°, 2×2 squares), C004 (0.3 c/°, 4×4 squares), C008 (0.6 c/°, 8×8 squares), C016 (1.2 c/°, 16×16 squares), C032 (2.4 c/°, 32×32 squares), C064 (4.79 c/°, 64×64 squares), and C128 (9.58 c/°, 128×128 squares). These conditions were tested in an online spelling task, which consisted of 18 trials each conducted on a 3×3 command interface. In addition to accuracy and information transfer rate (ITR), subjective measures regarding comfort, ocular irritation, and satisfaction were collected. Significant differences in performance and comfort were observed based on different stimulus spatial frequencies. Although all conditions achieved mean accuracy over 95% after 2.1 s of trial duration, C016 stood out in terms user experience. The proposed condition not only achieved a mean accuracy of 96.53% and 164.54 bits/min with a trial duration of 1.05s, but also was reported to be significantly more comfortable than the traditional C001 stimulus. Since both features are key for BCI development, higher spatial frequencies than the classical black-to-white stimulus might be more adequate for c-VEP systems. Hence, we assert that the spatial frequency should be carefully considered in the development of future applications for c-VEP-based BCIs.

## 1. Introduction

A brain-computer interface (BCI) is a technology that enables users to interact with the environment solely via their brain signal (Wolpaw and Wolpaw, 2012). In certain diseases where patients experience severe motor impairment, BCIs can offer a promising solution to restore the user's ability to engage with their surroundings. Previous applications of this technology include controlling devices for interaction, such as home automation systems, wheelchairs, or virtual keyboards (Iturrate et al., 2009; Kosmyna et al., 2016; Rezeika et al., 2018).

Among the different modalities for monitoring brain activity in BCI systems, electroencephalography (EEG) is the most commonly employed due to its portability, affordability, and suitable temporal resolution (Ramadan and Vasilakos, 2017). However, decoding user intentions from their brain activity is challenging since they are not directly reflected in the EEG, making it necessary to use different paradigms or tasks to detect them, i.e., control signals. Visual evoked potentials (VEPs) have found application as exogenous control signals in specific paradigms, as they reflect brain responses to external visual stimuli perceived by the user. Traditionally, steady-state VEPs (SSVEPs) have been extensively employed to encode commands that flicker at constant frequencies, inducing an oscillatory response in the EEG that can be used to decode target commands in real-time (Volosyak et al., 2020). Although SSVEP-based BCIs can achieve excellent accuracy, the monitor's refresh rate limits the number of commands to encode, and decoding within certain frequency bands (e.g., beta) can be challenging (Volosyak et al., 2011). Recently, code-modulated VEPs (c-VEPs) have emerged as a promising control signal to address these limitations (Bin et al., 2009; Martínez-Cagigal et al., 2021). However, despite their potential, c-VEP-based BCIs have yet to undergo extensive research and investigation.

BCI systems based on c-VEPs usually employ non-periodic binary time series (i.e., codes) to modulate the visual stimuli. This modulation is typically accomplished by associating black flashes with 0 values, and white flashes with 1 values within the sequence. Usually, codes with flat autocorrelation properties, such as maximal-length sequences (m-sequences), are used to encode commands using the circular shifting paradigm. In this paradigm, each stimulus flickers using different time-shifted versions of the same m-sequence (Martínez-Cagigal et al., 2021). This approach offers a remarkable benefit on calibration: the VEP elicited by the original m-sequence is used as the reference template, whereas the templates for the remaining commands are derived by temporally shifting this VEP. The real-time decoding of the intended command is achieved by correlating the EEG response and the calibrated templates. BCIs based on c-VEPs possess distinct advantages over other approaches such as event-related potential (ERP)-based BCIs, including reduced calibration time, and superior information transfer rate (ITR) (Martínez-Cagigal et al., 2021). Additionally, c-VEP systems exhibit comparable accuracy and speed to SSVEP-based BCIs, while being less susceptible to unrelated background EEG activity and typically imposing fewer restrictions on the number of available commands.

**Figure 1:**
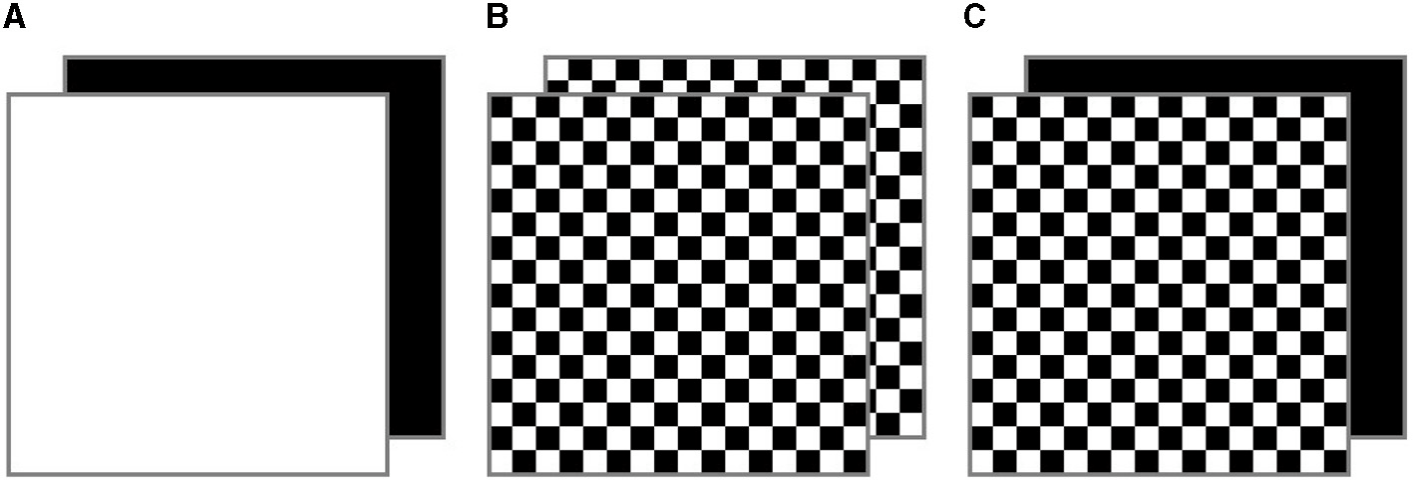
Pairs of alternating stimuli in the **(A)** single flickering, **(B)** checkerboard (CB), and **(C)** black background checkerboard (BB-CB) paradigms.

Although c-VEP-based BCIs are a relatively novel field of study, there have been previous investigations into the diversity of stimuli employed in these systems, albeit limited in number. For instance, some studies have explored the use of different sizes, colors, or distances between stimuli (e.g., Wei et al., 2016 or Nezamfar et al., 2016). However, the most commonly employed stimulus pairs are binary and monochromatic, referred to as single flickering paradigm, alternating intermittently between two colors (Figure 1A) (Martínez-Cagigal et al., 2021). By contrast, some studies have employed the checkerboard (CB) pattern instead of single flickering (e.g., Nezamfar et al., 2016, 2018; Isaksen et al., 2017). The CB pattern involves presenting a stimulus composed of alternating squares of different colors (e.g., white/black or red/green) that switch colors with each stimulus presentation (Figure 1B). Nevertheless, despite the incorporation of the CB pattern in previous c-VEP-based BCI studies, there is no previous comparison between this pattern and the traditional single flickering.

In previous c-VEP-based BCI proposals, the use of the CB pattern has been justified by potential improvements demonstrated compared to the standard single flickering pattern in SSVEP-based BCIs, such as enhanced performance or reduced visual fatigue (Waytowich et al., 2017; Ming et al., 2019). Specifically, these studies have manipulated the spatial frequency of the stimuli, which refers to the size of the squares within the stimulus, measured in cycles (pairs of squares of two alternative colors) per degree of visual angle (c/°). However, the effect of different spatial frequencies of the CB pattern on the performance of a c-VEP-based BCI remains unexplored (Martínez-Cagigal et al., 2021). Therefore, the findings from previous studies conducted in SSVEP-based BCIs that investigated the spatial frequency of the CB pattern will be detailed next. First, Waytowich et al. (2017) performed a gradual analysis of this variable (nine conditions from 0 c/° to 19.2 c/°) showing that the conditions with 0 c/° (single flickering) and 2.4 c/° demonstrated the highest performance. However, it was also observed that lower spatial frequencies were associated with higher perceived ocular irritation by the participants. Second, in order to improve the user experience, Ming et al. (2019) proposed a modified version of the CB pattern known as the black-background CB (BB-CB), where half of the squares remained black while the others alternated between white and black (Figure 1C). This paradigm achieved performance similar to the single flickering condition. Third, Ming et al. (2021) conducted a subsequent work to study the effect of spatial frequency using the BB-CB pattern with a gradual approach (nine conditions, from 0 c/° to 21.25 c/°), similar to Waytowich et al. (2017). This study yielded results consistent with those reported by Waytowich et al. (2017), indicating that the recommended condition presented a stimulus spatial frequency equal to 2.66 c/°. This condition showed no significant differences in performance compared to the single flickering paradigm, but a higher user preference and visual comfort. Finally, in a third study, Ming et al. (2023) demonstrated that presenting small gray squares on a static darker gray background (resembling the BB-CB paradigm but utilizing gray colors) yielded enhanced performance and subjective evaluation (including preference, comfort, and flicker perception) when compared to a condition where white and black squares alternated in the same location over a static gray background (similar to the CB paradigm, but with separate squares and a gray background). Finally, in a third study, Ming et al. (2023) showed improved performance and subjective evaluation (including preference, comfort, and flicker perception) with a condition similar to the BB-CB paradigm (light gray squares on a dark gray background) compared to a resembling CB paradigm condition (alternating black and white squares). Consequently, these results could suggest that the BB-CB paradigm may be more suitable than the CB paradigm.

It is noteworthy that both Waytowich et al. (2017) and Ming et al. (2021) yielded similar conclusions regarding spatial frequency for CB or BB-CB paradigms in SSVEP-based BCIs. Both studies indicated that the highest performance among the spatial frequencies (from 0 c/° to ~20 c/°) was observed for 0 c/° and ~2.5 c/°. These findings suggest that spatial frequency can influence the performance of SSVEP-based BCIs. Furthermore, they revealed that the subjective user experience (including comfort, visual irritation or preference) was negatively affected when using the single flickering condition. A usable system should not only demonstrate good performance but also be tailored to users' willingness to utilize it. Thus, it would be worthwhile to explore, for the first time, how the spatial frequency of the BB-CB paradigm [which was suggested to be even better than the standard CB paradigm; Ming et al. (2023)] could affect performance and subjective experience in a c-VEP-based BCI.

The aim of the present study is to explore the influence of stimulus spatial frequency using the BB-CB paradigm to control a c-VEP-based BCI. To accomplish this, eight different spatial frequencies will be evaluated (from 0 c/° to 9.58 c/°) in a nine-command interface during an online spelling task. To evaluate the impact of stimulus spatial frequency, both performance (accuracy and ITR) and subjective user experience measures (comfort, ocular irritation, and preference) will be analyzed.

## 2. Methods

### 2.1. Experimental BCI paradigm

**Figure 2:**
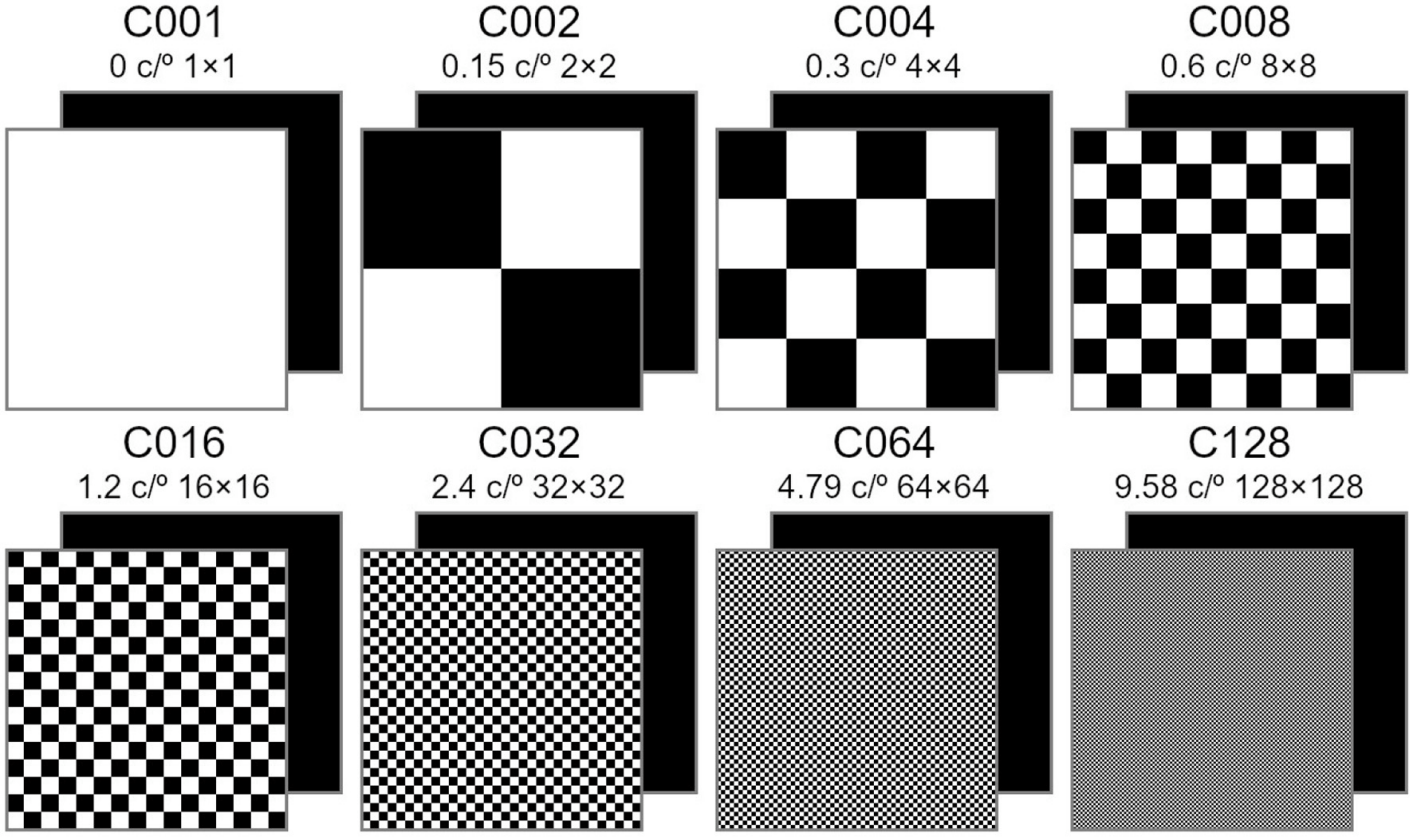
The black-background checkerboard (BB-CB) stimulus patterns with eight different spatial frequencies (c/°) and number of row and columns. All the stimulus patterns presented were paired with a flickering monochromatic black square.

To evaluate the impact of stimulus spatial frequency, the present study has examined eight conditions (spatial frequency and matrix size): C001 (0 c/° and 1×1), C002 (0.15 c/° and 2×2), C004 (0.3 c/° and 4×4), C008 (0.6 c/° and 8×8), C016 (1.2 c/° and 16×16), C032 (2.4 c/° and 32×32), C064 (4.79 c/° and 64×64), C128 (9.58 c/° and 128×128) (Figure 2). The selection of distinct spatial frequencies was based on conditions previously investigated by Waytowich et al. (2017) and Ming et al. (2021), both applied to SSVEP-based BCIs.

**Figure 3:**
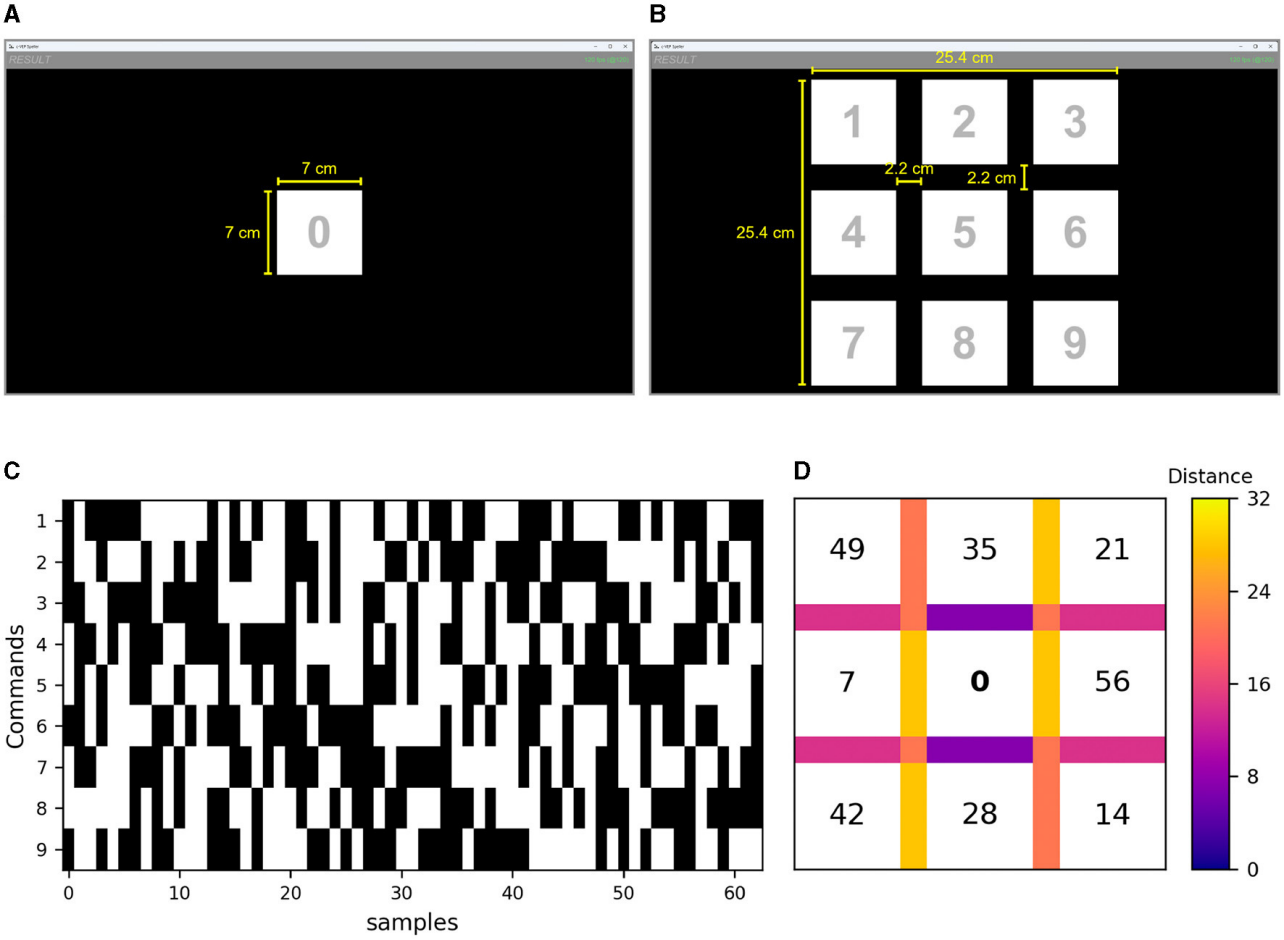
Screenshots of the layout for both stages of the experimental paradigm: **(A)** calibration and **(B)** online. Each command represented in the figure (i.e., the white squares with the number in the middle) is superimposed during the selection phase by the corresponding flickering stimulus of each experimental condition. The yellow lines and text were not displayed in the interface; they have been exclusively used in the figure to illustrate the dimensions (cm) of the respective elements. **(C)** Shifted versions of the m-sequence for each command available in the online stage. **(D)** Arrangement of lags for each command to minimize cross-talk between shifted m-sequences; i.e., lag = 0 denotes the original m-sequence, assigned to command “5”. The distances between neighboring commands are illustrated for horizontal, vertical, and diagonal connections. In the case of diagonal neighbors, the average distance from both diagonals is displayed. Additionally, the minimum and maximum distances in the layout are indicated as solid white lines in the colorbar.

Each condition consisted of two BCI stages: (i) a calibration stage to adapt the system for each user by training a customized model, and (ii) an online stage where users actually controlled the system. In the calibration stage, the layout presented to users consisted of a single command encoded with the original m-sequence (Figure 3A). In the online stage, the layout comprised 9 distinct commands, arranged within a 3×3 matrix (Figure 3B). In this stage, users received feedback using the customized model trained in the calibration phase. The other visual parameters remained similar between stages. The interface's background color was black. Commands were gray numbers (0 in the calibration task, and 1-9 in the online task) surrounded by a white square measuring 7×7 cm (6.7×6.7° at 60 cm). These numbers were overlaid with flickering stimuli (the m-sequence) that required users' attention. The flickering stimuli were the same size as the white squares with the numbers, and they also were opaque, causing the number to become non-visible once the selection phase began. The distance between the adjacent edges of each stimulus was 2.2 cm (2.1° at 60 cm) both horizontally and vertically. Each matrix's command flashed according to time-shifted versions of a single m-sequence. A 63-bit m-sequence was generated by employing a linear feedback shift register (LFSR) with the primitive polynomial *x*^6^+*x*^5^+1 and an initial state of 111110. For a more comprehensive exposition on the methodology employed in generating the m-sequence, kindly refer to Martínez-Cagigal et al. (2021). In order to encode the nine distinct commands, the original m-sequence underwent a temporal shift of θ_*i*_ = τ*i* samples, where τ = 7 and *i* = 0, 1, …, 8. A genetic algorithm (GA) was used to minimize cross-talk between shifted m-sequences, ensuring that commands with consecutive lags were not placed adjacently. The pseudocode for the GA is provided in the Supplementary material. The final arrangement of commands, as well as the shifted versions of the m-sequence for each command, are illustrated in Figures 3C, D.

The presentation rate of the m-sequence events was set to 120 Hz (one sample of the m-sequence each 8.33 ms), resulting in each cycle of 63 bits lasting 525 ms (8.33 ms×63 samples). In order to make a selection, participants were required to focus their attention on the target for 8 complete cycles of the m-sequence (4.2s, 525 ms×8 cycles). In the online stage, the other commands in the matrix flashed simultaneously with their respective lagged versions of the m-sequence. A 3-s pause was implemented between selections (i.e., trials) in both stages. During this pause in the online stage, the selected command was highlighted in green for 1 s to provide feedback to the user.

### 2.2. Data acquisition and signal processing

All aspects of BCI operation were controlled by the MEDUSA^©^ software ecosystem (Santamaría-Vázquez et al., 2023) running on a PC Intel Core i7-7700 (3.6 GHz, 32 RAM). The experiment was displayed in a monitor Keep Out XGM24F+ (144 Hz, 16:9 ratio, 52.64×29.61 cm, 23.8 in, 60.4 cm, 1920×1080 pixels) connected via HDMI. The refresh rate of the screen was set to 120 Hz. The experimental conditions were developed in a Unity-based application that communicates with MEDUSA^©^ via TCP/IP sockets to ensure exact synchronization between stimuli onsets and EEG registering (Santamaría-Vázquez et al., 2023). EEG data were recorded by a g.USBamp (g.Tec, Guger Technologies, Austria) amplifier with 16 channels and a sampling rate of 256 Hz. Fifteen active electrodes were placed at predefined locations on the EEG-cap in accordance with the 10/10 international system: F3, Fz, F4, C3, Cz, C4, CPz, P3, Pz, P4, PO7, PO8, Oz, I1, and I2. All channels were referenced to the right earlobe and grounded to position AFz.

EEG signals were processed in real-time using a pipeline mainly based on the reference processing for circular shifting (Martinez-Cagigal et al., 2023). A pre-processing step consisting on a series of 7-th order infinite impulse response Butterworth filters was employed to enable real-time processing. First, a notch filter at 50 Hz was applied to remove power line interference. Then, a filter bank consisting of three bandpass filters was used to improve the differentiation between natural brain activity, such as alpha band activity (associated with tiredness), and responses triggered by the c-VEP stimuli (Gembler et al., 2020). The initial filter covered a wide frequency range of 1–60 Hz, capturing all frequency bands. The second filter focused on the beta and gamma frequency bands (12–60 Hz), while the third filter specifically targeted the gamma band (30–60 Hz) (Martinez-Cagigal et al., 2023). The upper cutoff of 60 Hz was chosen based on the maximum frequency that can be represented in the EEG due to the refresh rate of the monitor (120 Hz). On the other hand, the lower cutoff of 1 Hz in the first filter accounted for the delta and theta frequency bands, which also contain important information related to the repetition of the stimuli used (Martínez-Cagigal et al., 2021). After the filtering process, canonical correlation analysis (CCA) was employed in each trial to decode the user's intended target command in real-time for an online task.

During the calibration phase, the user is required to focus on a command encoded with the original m-sequence, without any temporal lag, for a duration of *k* cycles. This process yields a pre-processed EEG signal $X\in {\mathbb{R}}^{N_{f},k,N_{s},N_{c}}$, where *N*_*f*_ = 3 denotes the number of filters in the bank, *N*_*s*_ represents the number of samples within a cycle, and *N*_*c*_ indicates the number of EEG channels. Notably, the number of samples per cycle is calculated as *N*_*s*_ = ⌈*f*_*s*_·*L*/*f*_*m*_⌉, where *f*_*s*_ = 256 Hz denotes the sampling rate of the EEG, *L* = 63 represents the length of the m-sequence, and *f*_*m*_ = 120 Hz is the presentation rate. For each filter *f* in the filter bank, a multi-channel VEP response ${\hat{X}}_{f}\in {\mathbb{R}}^{N_{s},N_{c}}$ is obtained by averaging across the cycles. Subsequently, a canonical correlation analysis (CCA) is trained to determine the linear projections ***W***_*a*_ and ***W***_*b*_ that maximize the correlation between the projected versions of two signals ***A*** and ***B***, thus optimizing:


(1) $$\begin{array}{l} \underset{W_{a},W_{b}}{\max}\frac{W_{a}^{T}A^{T}BW_{b}}{\sqrt{W_{a}^{T}A^{T}AW_{a}\cdot W_{b}^{T}B^{T}BW_{b}}}, \end{array}$$

where $A\in {\mathbb{R}}^{kN_{s},N_{c}}$ represents the concatenated version of ***X*** for a given filter, and $B\in {\mathbb{R}}^{kN_{s},N_{c}}$ is the multi-channel VEP ${\hat{X}}_{f}$ repeated *k* times to match the dimensions. Upon training the CCA, spatial filters $W_{a}\in {\mathbb{R}}^{N_{c},N_{c}}$ and $W_{b}\in {\mathbb{R}}^{N_{c},N_{c}}$ are obtained. However, only the first column of ***W***_*b*_, denoted as ***ω***_*b*_, is used as a spatial filter to project the averaged response (i.e., ${\hat{X}}_{f}\cdot w_{b}$) and generate the main template ${\tilde{x}}_{f0}\in {\mathbb{R}}^{N_{s},1}$. Templates for the remaining commands ${\tilde{x}}_{fi}$ are generated by circularly shifting ${\tilde{x}}_{f0}$ based on the associated temporal lag θ_*i*_ = τ*i* for each command, where τ = 7 and *i* = 0, 1, …, 8. This procedure is repeated for each filter *f*, resulting in three distinct sets of templates. Additionally, the standard deviation σ_***A***_ of the signal matrix *A* is computed for each channel. Artifacts are identified within a given cycle if the standard deviation of that specific epoch exceeds three times the value of σ_***A***_. Only epochs that do not exhibit artifacts in any of the channels are used for training the model (Martinez-Cagigal et al., 2023).

During the online mode, at the conclusion of each trial, the identical pre-processing stage is implemented to obtain the matrix $Z_{\text{test}}\in {\mathbb{R}}^{N_{f},k_{t},N_{s},N_{c}}$, where *k*_*t*_ represents the number of cycles in the test trial. For each filter *f*, the signal is averaged across cycles and projected using the trained spatial filter ***ω***_*b*_, resulting in the test response ${\tilde{z}}_{f}\in {\mathbb{R}}^{N_{s},1}$. The test response is then compared with all the templates corresponding to filter *f*, yielding a vector ***r***_*f*_ comprising the Pearson's correlation coefficients for each command. Finally, the correlations are averaged across the filter bank, and the selected command corresponds to the one associated with the maximum coefficient, i.e., $y=\arg\underset{i}{\max}\frac{1}{f}\underset{f}{\sum }r_{f}$ (Martinez-Cagigal et al., 2023).

### 2.3. Participants and procedure

The study has involved 16 participants (aged 29.63 ± 4.06, 11 males, 5 females) with normal or corrected-to-normal vision and varying experience in the use of these systems (ranging from users with more than 5 sessions to beginners). The protocol was approved by the local ethics committee and met the standards of the Helsinki Declaration. The experiment was conducted in a single session lasting approximately 75 min. Upon arrival at the laboratory, participants were given an explanation of the task their would carry out, signed the informed consent form, and the necessary equipment was set up. The user performed the tasks while comfortably seated in a distraction-free room, where only the researcher and the participant were present.

The design used was intrasubject, also known as repeated measures design, so all participants tested the eight different experimental conditions that varied in the spatial frequency of the stimuli. As it was explained in Section 2.1, each condition consisted of both calibration and online stages. The calibration stage comprised two runs of 15 trials each (i.e., 30 selections), while the online task consisted of one run of 18 trials. There was a short break between runs, and its length varied based on the user's preference. During the calibration task, participants were instructed to pay attention to the single stimulus presented on the interface. However, in the online task, stimuli were presented with the 3×3 matrix and participants were required to select the numbers following the row-major order (from 1 to 9) twice. After completing the online stage, participants were required to respond to a questionnaire to assess their subjective experience while controlling the system. The user moved on to the next experimental condition only after completing the questionnaires related to the previous condition. The order of conditions was counterbalanced, so each condition was evenly distributed among the participants (as there were 16 participants, each condition was used twice in each position), to mitigate any potential undesirable effects such as learning or fatigue.

### 2.4. Evaluation

#### 2.4.1. Performance

To evaluate the impact of stimulus spatial frequency on the calibration and online tasks, two parameters were utilized: (i) accuracy (%), which measures the percentage of correctly classified selections out of the total predicted selections; and (ii) ITR (bits/min), which provides an objective measurement of the system's information rate and is calculated based on the traditional formula presented in Wolpaw et al. (1998). The ITR takes into account the accuracy (*P*), the number of commands available in the interface (*N*), and the number of selections per minute (*Q*):


(2) $$\begin{array}{l} ITR=Q\cdot \left[\log_{2}N+P\log_{2}P+\left(1-P\right)\log_{2}\left(\frac{1-P}{N-1}\right)\right] \end{array}$$

The performance measures (accuracy and ITR) in the online tasks were examined at two levels. Firstly, for each metric, the Friedman test was employed to explore whether there was a main effect of the spatial frequency of the stimuli or the number of elapsed cycles on the system's performance. In order to assess the main effect of spatial frequency, the average value across the eight cycles was calculated. Likewise, to evaluate the main effect of cycles, the average score across the eight conditions was obtained. In cases where a significant main effect was identified, multiple Wilcoxon signed-rank tests were conducted to determine which specific conditions or cycles showed differences. In these subsequent comparisons, the *p*-values were adjusted using the Benjamini-Hochberg procedure to control the false discovery rate and minimize type I errors (Benjamini and Hochberg, 1995). Secondly, the same analysis procedure was carried out for each of the cycles. The Friedman test was used to determine which specific cycles showed differences among the conditions. Subsequently, in those cycles where the Friedman test found significant differences, multiple *post-hoc* Wilcoxon signed-rank tests were employed to identify the specific conditions that differed for that particular cycle.

#### 2.4.2. Brain responses

EEG signals were examined in order to investigate how brain responses are affected by different spatial frequencies of the stimuli. To achieve this, a grand average was calculated across all participants and cycles for each condition using the calibration data, resulting in amplitude values within the EEG signal for each specific condition. The Oz channel was chosen for this analysis because it has been identified as the most informative in reflecting the primary visual cortex activity, located in the occipital lobe (Bin et al., 2009; Wolpaw and Wolpaw, 2012). Additionally, the Pearson product-moment correlation coefficients were calculated to determine the interdependence between each pair of conditions throughout the duration of the cycle.

#### 2.4.3. Subjective items

After completing the online task for each condition, participants were asked to rate three items on a scale from 0 to 10 (“very low” and “very high”, respectively): (i) comfort level during interface operation, (ii) ocular irritation experienced while controlling the interface, and (iii) level of general satisfaction. Three ANOVAs were conducted to investigate the impact of stimulus spatial frequency on these variables. In cases where significant effects were identified in the ANOVA, multiple *post-hoc* repeated measures *t*-test were conducted; the *p*-values were adjusted using the Benjamini-Hochberg procedure. Additionally, at the end of the session, participants were asked to indicate their preferred condition. To evaluate potential significant differences among the spatial frequencies in terms of participants' preferences, a binomial test was conducted for each condition to determine if it was chosen significantly above or below the chance level.

## 3. Results

### 3.1. Performance

**Figure 4:**
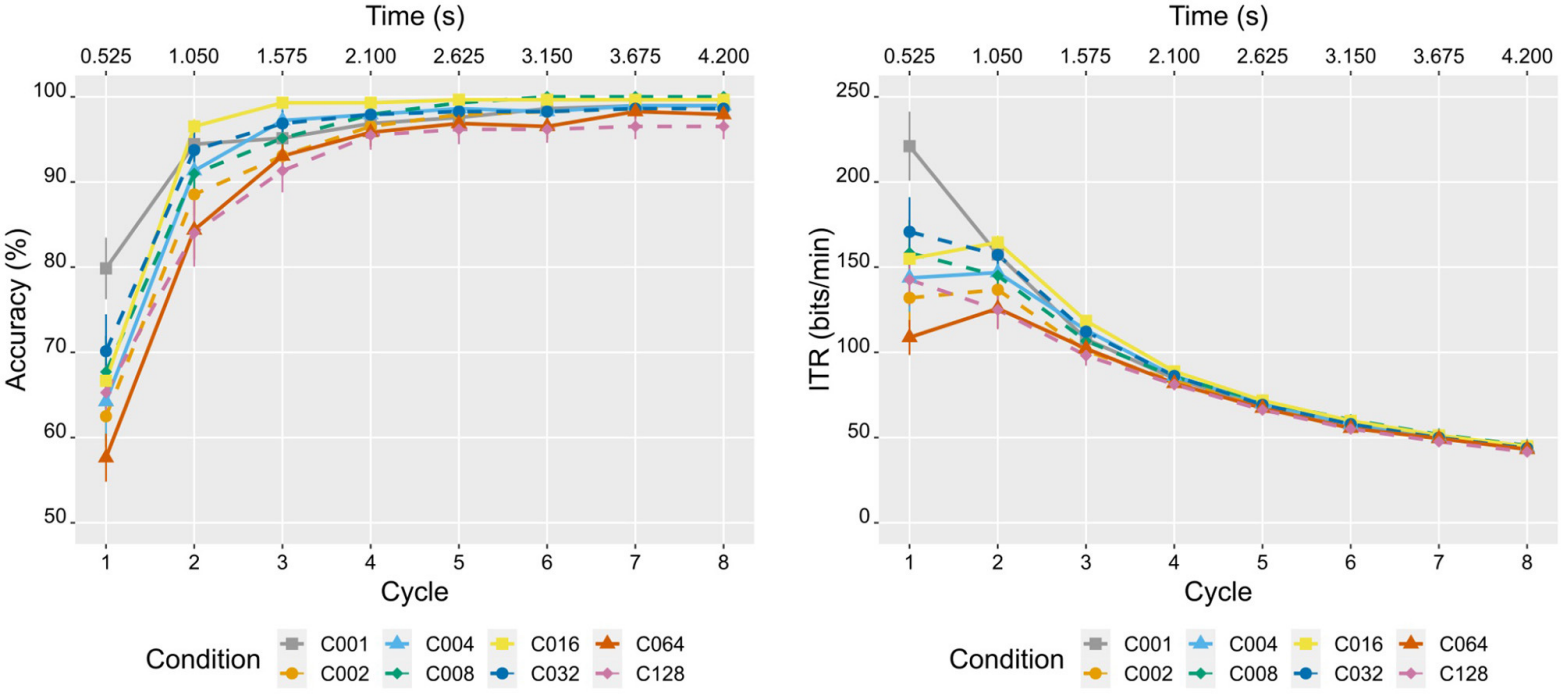
Performance results (mean ± standard error) for each of the experimental conditions as a function of time (s) and the number of cycles elapsed in the online task for the variables accuracy **(left)** and information transfer rate (ITR, **right**). Because there were nine commands available in the interface, the theoretical accuracy level was equal to 1/9 (i.e., 11.11%).

The average performance in the online task of all conditions surpassed 95% accuracy within just 4 cycles (2.1 s), with even 84% accuracy achieved in half the time (2 cycles, 1.05 s) (Figure 4). This high accuracy achieved in such a short duration is also reflected in the high levels of ITR. On average, all conditions yielded ITRs above 120 bits/min with at least 84% accuracy (2 cycles). Condition C001 even reached an average ITR of 220 bits/min with 80% accuracy using a single cycle (525 ms). Although the performance of all conditions was commendable, a more in-depth analysis was conducted to examine potential differences among the experimental conditions (varying in stimulus spatial frequency) in terms of accuracy and ITR. For the sake of completeness, unfolded performance results (accuracy and ITR) for each participant and condition are detailed in the Supplementary material.

In reference to accuracy, the Friedman test revealed a main effect of condition [χ^2^_(7)_ = 21.384, *p* = 0.003, *W* = 0.191] and cycle [χ^2^_(7)_ = 101.856, *p* < 0.001, *W* = 0.909]. First, despite finding a main effect in relation to the condition factor, none of the *post-hoc* comparisons (Wilcoxon signed-rank tests) between conditions have been significant. Second, the cycle factor has shown significant differences among all combinations of cycles (*p* < 0.05, with higher performance observed in the later ones), except between cycles 5 and 6 (*W*^+^ = 10.5, *p* = 0.336, *r* = 0.034), and 7 and 8 (*W*^+^ = 2.5, *p* = 1, *r* = 0.028). Third, it is interesting to check in which specific cycles the accuracies differed between conditions. The Friedman test showed significant differences between conditions in cycle 1 [χ^2^_(7)_ = 22.75, *p* = 0.002, W = 0.203], cycle 2 [χ^2^_(7)_ = 15.227, *p* = 0.033, W = 0.136], and cycle 3 [χ^2^_(7)_ = 17.998, *p* = 0.012, W = 0.161]. However, according to multiple *post-hoc* analyses (Wilcoxon signed-rank tests), only the following significant differences were found (the first of each pair provided the best result): in cycle 1, C001 vs. C002 (*W*^+^ = 117.5, *p* = 0.017, *r* = 0.538), C064 (*W*^+^ = 120, *p* = 0.017, *r* = 0.622) and C128 (*W*^+^ = 98, *p* = 0.044, *r* = 0.452).

Regarding ITR, the Friedman test showed a significant main effect for both the condition [χ^2^_(7)_ = 22.959, *p* = 0.002, *W* = 0.205] and the cycle factors [χ^2^_(7)_ = 109.396, *p* < 0.001, *W* = 0.977]. First, regarding the main effect of the condition factor, significant differences have been found in ITR between the following pairs of conditions (the first of each pair provided the best result): C001 vs. C002 (*W*^+^ = 122, *p* = 0.027, *r* = 0.45) and C064 (*W*^+^ = 130, *p* = 0.012, *r* = 0.52), C016 vs. C064 (*W*^+^ = 99, *p* = 0.027, *r* = 0.403), and C032 vs. C064 (*W*^+^ = 113, *p* = 0.027, *r* = 0.42). Second, the cycle factor has shown significant differences among all combinations of cycles (*p* < 0.05, with lower ITR observed in the later cycles), except for cycles 1 and 2 (*W*^+^ = 88, *p* = 0.323, *W* = 0.087). Third, it should be verified during which cycles there were differences between the conditions. The Friedman test showed significant differences between conditions in cycle 1 [χ^2^_(7)_ = 22.75, *p* = 0.002, W = 0.203], cycle 2 [χ^2^_(7)_ = 15.227, *p* = 0.033, W = 0.136], and cycle 3 [χ^2^_(7)_ = 17.998, *p* = 0.012, W = 0.161]. However, the pairwise analyses only revealed the significant differences in cycle 1 (the first of each pair provided the best result): C001 vs. C002 (*W*^+^ = 118.5, *p* = 0.014, *r* = 0.538), C064 (*W*^+^ = 120, *p* = 0.014, *r* = 0.622) and C128 (*W*^+^ = 98, *p* = 0.044, *r* = 0.452).

### 3.2. Brain responses

**Figure 5:**
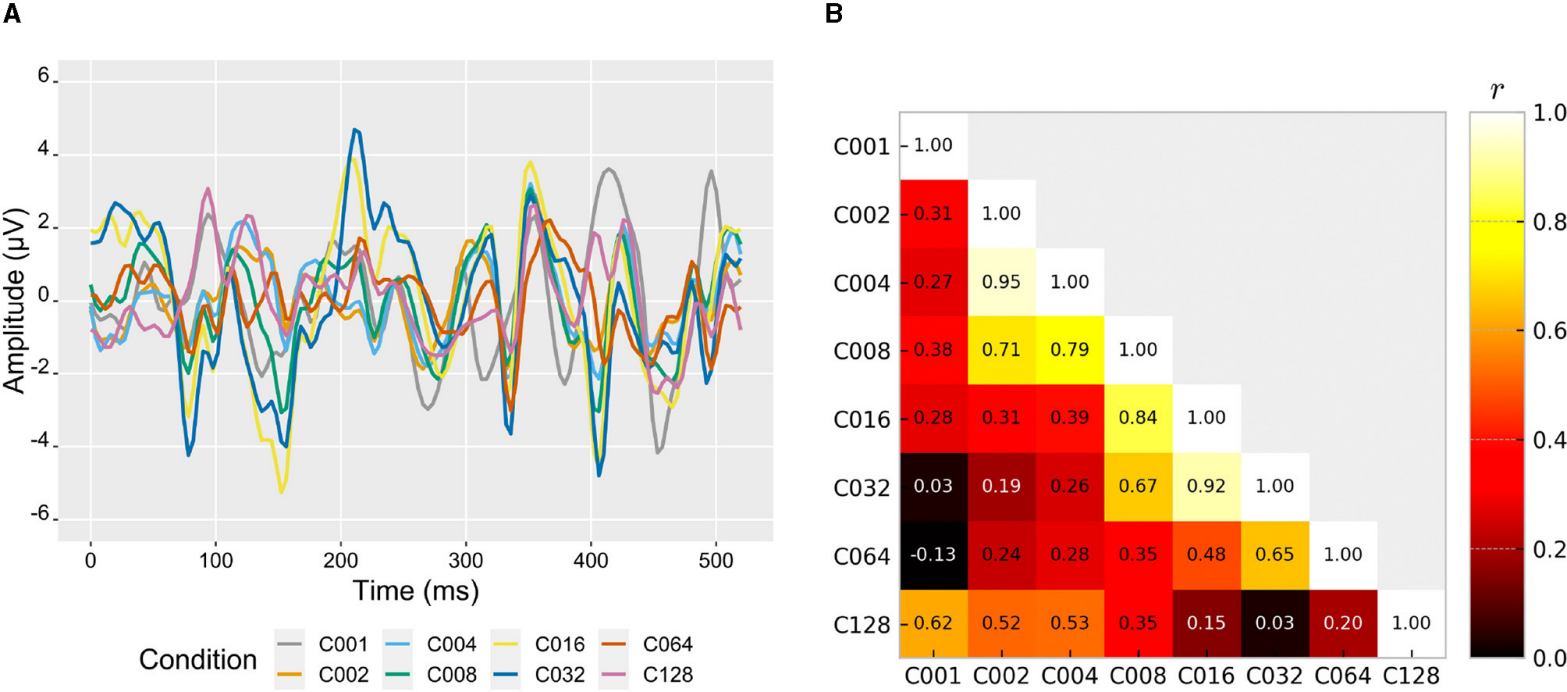
**(A)** Grand-averaged visual evoked potentials (VEPs) of all users to the eight different spatial frequency conditions. These VEPs were extracted from calibration epochs over the Oz channel. **(B)** Pearson product-moment correlation coefficient *r* between the depicted VEPs among all different conditions.

In Figure 5A, the grand-averaged VEPs of the participants for each condition at channel Oz can be observed during a cycle duration (525 ms). First of all, it is interesting to note that despite using the same m-sequence for all conditions, there are conditions whose EEG signal was notably different (e.g., C016 and C128), while other conditions exhibit a similar EEG signal pattern (e.g., C016 and C032). The individual VEP responses for all participants are depicted in the Supplementary material, revealing a remarkable resemblance across conditions among the participants, which expose the exogenous nature of the c-VEP responses. To facilitate the exploration of similarities between conditions, a correlation analysis was conducted on different pairs of conditions (Figure 5B). The results indicated that conditions with adjacent spatial frequencies (e.g., C002–C004, C004–C008, C008–C016, C016–C032) tended to exhibit stronger correlations. However, C128 showed a higher correlation with conditions that had lower spatial frequencies (C001, C002 and C004). These findings may suggest that the similarity in VEPs could also indicate a similarity in how users perceive stimuli from different conditions.

### 3.3. Subjective items

**Figure 6:**
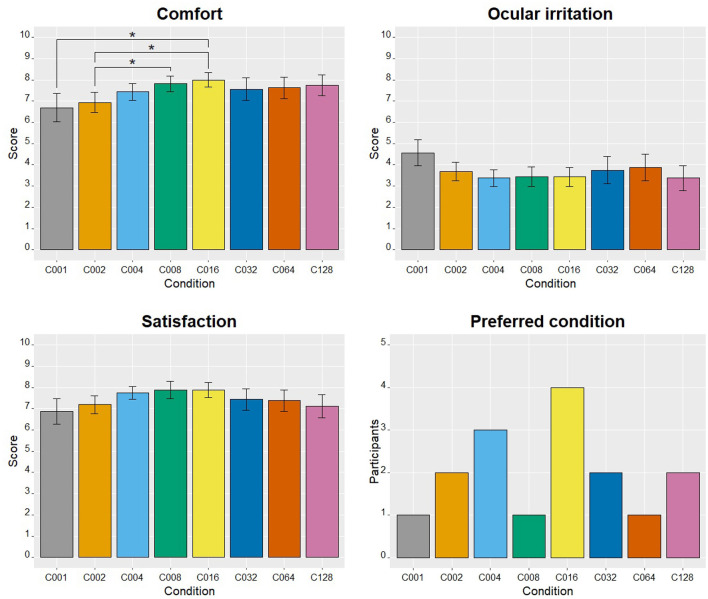
Results obtained in the subjective questionnaires (mean ± standard error) regarding comfort, ocular irritation, and satisfaction, as well as the histogram related to the number of participants who selected each spatial frequency stimulus as their preferred one. For the variables comfort, ocular irritation, and satisfaction, The presence of significant differences between conditions (*p* < 0.05) has been denoted with an asterisk. False discovery rate was corrected using the Benjamini-Hochberg procedure for multiple comparisons.

Figure 6 shows the average scores given by users for comfort, ocular irritation, and satisfaction items for each condition. To verify if spatial frequency had an effect on these variables, three one-way repeated measures ANOVA tests were conducted for each of these variables. Regarding comfort, the analysis showed a significant main effect of the condition factor [*F*_(3.55, 53.19)_ = 2.838; *p* = 0.039, $\eta_{p}^{2}$ = 0.159]. Therefore, it can be stated that the stimulus spatial frequency has influenced the comfort of the system during its control. Specifically, the conditions that exhibited significant differences in the multiple comparisons were as follows (the first of each pair provided the highest comfort level): C016 vs. C001 (*p* = 0.043, *d* = 0.831) and C002 (*p* = 0.019, *d* = 1.065), as well as C008 vs. C002 (*p* = 0.043, *d* = 0.854). On the contrary, no significant differences were found in the ANOVAs regarding ocular irritation [*F*_(3.32, 49.8)_ = 1.598; *p* = 0.198, $\eta_{p}^{2}$ = 0.096] or satisfaction [*F*_(3.39, 50.81)_ = 1.055; *p* = 0.382, $\eta_{p}^{2}$ = 0.066]. Therefore, these results suggest that neither ocular irritation nor satisfaction were significantly influenced by the stimulus spatial frequency. Finally, none of the binomial tests conducted for each condition showed any of them being selected significantly above or below the chance threshold.

## 4. Discussion

### 4.1. General discussion and related literature

The results of this study seem to indicate that stimulus spatial frequency has a clear effect on the performance (accuracy and ITR) of the c-VEP-based BCI, as well as on the users' comfort during its control. Indeed, based on the variables studied, two experimental conditions could be highlighted: C001 (0 c/°) and C016 (1.2 c/°). However, the choice of one condition or another will depend on the specific characteristics and needs of the system being controlled. C001 achieved high accuracy with just one cycle, but was later surpassed by C016 (although not significantly). Besides, users rated C001 as significantly less comfortable than C008 and C016. The comfort variable is also relevant since these systems are sometimes intended for everyday use by patients for long periods. Secondly, although there were no significant differences in ocular irritation or satisfaction, C001 also presented the most unfavorable scores for both variables. Furthermore, it also appears clear that C002, C064 and C128 yielded poorer performance and worse subjective ratings for comfort, particularly in the case of C002. Therefore, these conditions (C002, C064 and C128) do not seem to be the most recommended based on the results obtained by the present study.

Next, we will proceed to compare the performances obtained in this study against the two previous studies that evaluated stimulus spatial frequency using an SSVEP-based BCI (Waytowich et al., 2017; Ming et al., 2021) (Table 1). On one hand, Waytowich et al. (2017) highlighted two conditions as the most suitable: 0 c/° and 2.4 c/°. The 0 c/° condition maintained a consistent accuracy of 97.7% from 3.5 s to 6s. In contrast, the 2.4 c/° condition reached its peak accuracy of 85.1% at 2.5 s and exhibited a gradual decline until the end of the 6-second period. Notably, the 2.4 c/° condition demonstrated the highest ITR among all conditions, achieving 45.3 bits/min at 1.5s, while the peak ITR for the 0 c/° condition was 35.7 bits/min at 2s. On the other hand, Ming et al. (2021) assessed the online performance of the condition that appeared to offer the best results following those obtained in the offline stage, characterized by a spatial frequency of 2.13 c/°. This condition showcased an excellent accuracy of 99.1% and an ITR of 124 bits/min (1.5 s). Finally, in our case, our peak ITR was achieved for C001 in cycle 1 (221.02 bits/min), with an average accuracy of 79.86% (1 cycle, 525 ms). However, despite showing a high ITR, there may be systems where errors incur a high cost, so it is advisable to sacrifice some ITR to increase the accuracy of the system. In that case, C016 could be highlighted as it reached an ITR of 164.54 bits/min with 96.53% accuracy (2 cycles, 1.05 s), or a more conservative option with 99.31% accuracy and 118.49 bits/min (3 cycles, 1.575 s). Therefore, the proposed system has demonstrated an adequate performance that is comparable to that observed in Ming et al. (2021), and higher to that observed in Waytowich et al. (2017).

**Table 1:** Performance comparison with previous SSVEP-based studies.

**References**	**Spatial frequency (c/°)**	**Accuracy (%)**	**Time (s)**	**ITR (bits/min)**
Waytowich et al. (2017)	0	97.7	3.5	~32^a^
	2.4	~78^a^	1.5	45.3
Ming et al. (2021)	2.13	99.1	1.5	124
Present study	0 (named C001)	79.86	0.525	221.02
	1.2 (named C016)	96.53	1.05	164.54
	2.4 (named C032)^b^	96.88	1.575	118.49

a The results of these metrics were obtained approximately from the figures reported in the articles since the exact values were not reported.

b Results from C032 are depicted to offer a fair comparison with respect to the spatial frequency proposed by the previous SSVEP-based studies.

The findings regarding performance and spatial frequency in the present study are also consistent with previous research conducted using an SSVEP-based BCI. In the works of Waytowich et al. (2017) and Ming et al. (2021), the highest performances (accuracy and/or ITR) were achieved with 0 c/° and ~2.3 c/°. In our case, the results exhibited a notable degree of similarity, with the exception being that our most favorable conditions were C001 (0 c/°), and C016 (1.2 c/°) instead of C032 (2.4 c/°), which exhibit the spatial frequency closest to ~2.3 c/°. It is worth noting that our layout was similar to the one used by Ming et al. (2021): nine commands arranged in a square 3×3 matrix, with stimuli of an equivalent size (7×7° in theirs, 6.7×6.7° in ours) and spacing between commands as well (1.4° in theirs, 2.1° in ours). Hence, the difference in optimal spatial frequency may be due to specific properties of the SSVEP and c-VEP signals. It would be highly recommended for future research to delve into the reasons behind these differences among signal types. Such investigation would enable the development of more precise proposals in the future, supported by a solid theoretical foundation.

The analyses related to the brain responses have shown interesting results that could provide insights for new proposals related to the characteristics of visual stimuli in c-VEP-based BCIs. Firstly, although the used m-sequence for stimuli presentation is the same for all conditions, the associated VEPs for each of them have exhibited notable differences. These variations might account for the disparities in performance and user comfort observed across different conditions. Furthermore, in relation to each condition, the averaged calibration epochs have shown little deviation across trials and participants. This pattern reflects a strong exogenous response that presents a high inter-trial and inter-subject robustness of the templates. Several previous studies have tried to model c-VEP responses as a linear superposition of the reactions to individual events or flashes, e.g., to reduce calibration time (Thielen et al., 2021), propose processing pipelines (Nagel and Spüler, 2019), or handcraft optimal codes (Yasinzai and Ider, 2020). The observed dissimilarity in brain responses among different conditions suggests that spatial frequency also plays an important role in the elicitation of such responses. Consequently, the incorporation of additional stimulus-derived parameters into this c-VEP modeling could potentially constitute a promising avenue for future research endeavors. Secondly, Figure 5B also showed that conditions with similar spatial frequencies had higher EEG signal amplitude correlation (from C002 to C064, adjacent correlations were *r*≥ 0.65). This suggests that similar visual stimuli lead to similar EEG patterns. However, C128 exhibited a higher correlation with conditions characterized by lower spatial frequencies. This could indicate that if the spatial frequency is too high, the small squares cease to be perceived individually but rather as a whole (similar to C001, which has the highest correlation). In this respect, the global perception of the small white-and-black squares would be perceived as a general shade of gray. This could have resulted in the stimuli of C128 being perceived with lower luminosity contrast (gray compared to white, on a black background). This is in line with the previous scientific literature that claims that the primary visual cortex is more responsive to high-contrast stimuli (Wandell et al., 2007). As a consequence, stimuli with lower contrast, such as gray/black vs. white/black, can result in a reduced signal-to-noise ratio (SNR) and, therefore, decreased classification accuracy (Ladouce et al., 2022). To summarize, the VEP of both conditions (C001 and C128) exhibits a correlation of *r* = 0.62, indicating perceptual similarity in the pattern. However, the luminosity contrast may have an impact on SNR, also affecting the accuracy. While the influence of spatial frequency on VEPs in reaction to identical m-sequences has been evidenced, it is our belief that the relationship between brain responses and performance is still not clear and should be studied further in the future to uncover the mechanisms at play.

Finally, the results regarding subjective measures will be discussed, contextualizing them whenever possible with previous studies on spatial frequency. Firstly, in terms of comfort, similar results to Ming et al. (2021) were obtained, where the C001 condition showed the lowest score, presenting significant differences compared to C008 and C016. It would be interesting for future studies to investigate the underlying reasons for these differences, such as ease of attention focus, size of the illuminated area, system performance, etc. Secondly, both Waytowich et al. (2017) and Ming et al. (2021) demonstrated that visual irritation and flickering perception, respectively, were more bothersome in the 0 c/° condition. Although not significant, our results have shown a trend toward increased ocular irritation for C001, the condition with the highest contrast luminosity. In the BB-CB-based conditions (C002–C128), the stimulus was only illuminated on 50% of the surface with squares of different sizes. Hence, this trend regarding ocular irritation was consistent with previous literature (Gembler et al., 2020; Ladouce et al., 2022; Martinez-Cagigal et al., 2023), and it could be attributed to an increased activation of the LMS (luminance) postreceptoral pathway (Gentile and Aguirre, 2020). Consequently, this effect may contribute to the lower comfort levels observed for C001, and it should be considered for situations in which a user has to control the application for an extended period of time. Thirdly, regarding the absence of significant differences in satisfaction, it is possible that all conditions achieved similar high scores as a result of utilizing 8 cycles, which led to a ceiling effect where all conditions nearly reached 100% accuracy. However, a different number of cycles may alter the satisfaction measure. For instance, if only 1 cycle had been used, the satisfaction results might have favored C001 due to: (i) its better performance with that number of cycles; or (ii) because the test duration of each condition was too short to induce ocular irritation. Finally, concerning the preferred condition, the analyses did not reveal anything statistically significant; however, it is worth mentioning that 25% of the participants chose C016, which aligns with the positive results observed in the other subjective variables, as well as the performance.

### 4.2. Limitations and future studies

Our findings reveal that the performance and user experience were indeed impacted by the stimuli spatial frequency in controlling a c-VEP-based BCI. Nevertheless, certain aspects warrant careful consideration and could be further explored in future studies. First, eight experimental conditions were tested, so each condition was controlled for a relatively short period of time (approximately 7 min per condition, including calibration and online stages), possibly deviating from the everyday use experienced by users relying on these systems. Fatigue-related effects such as ocular irritation may not be observable until the application is used for an extended period. Thus, a future research line could be focused on investigating the preferred conditions and conduct a more exhaustive and protracted usability analysis. Second, it would be relevant to verify the obtained results among users who represent the target population for these interfaces, such as individuals affected by conditions like amyotrophic lateral sclerosis, whose cognitive or visual abilities may differ from those of the sample used in the present study (McFarland, 2020). Also, these individuals would want to control practical applications (e.g., a speller or a home automation system). Thus, the proposed system should be adjusted for such applications. For example, in the present study, the commands to be selected (i.e., numbers from 1 to 9) were only shown before the trial began. In a continuous control system, those commands would never be displayed, and therefore, the interface would need to be adapted. Third, the used system consistently employed a fixed number of cycles, whereas it is possible that adequate system control could be achieved with fewer cycles (e.g., C016 achieved an average accuracy of 99.31% with only 3 cycles, 1.575 s). Therefore, future proposals could consider incorporating the use of dynamic stopping methods to improve system efficiency (Thielen et al., 2021). Fourth, it may be important to explore how the stimulus spatial frequency relates to other visual variables. For example, it could be interesting to explore the effect of spatial frequency under different color combinations instead of black/white (Nezamfar et al., 2016), the use of real-world backgrounds to interact with environmental elements (Riechmann et al., 2016), or the effect of stimuli with lower luminosity to reduce visual fatigue (Ming et al., 2023). It is also crucial to note that, in the BB-CB paradigm, manipulating the stimulus spatial frequency, while keeping the size constant, also modifies the number of white squares appearing over the black background. Hence, investigating the potential interaction effect between spatial frequency and overall stimulus size (e.g., smaller sizes may benefit from a higher spatial frequency than larger sizes) would be also interesting.

## 5. Conclusions

To our knowledge, this is the first work that has evaluated the effect of stimulus spatial frequency on a cVEP-based BCI speller. Our results determined that stimulus spatial frequency presented a significant impact on performance and user comfort. Specifically, two conditions utilized in the study should be highlighted: C001 (0 c/°) and C016 (1.2 c/°). On one hand, C001 stood out with a high ITR (221.02 bits/min) at very short selection times (525 ms), but with an accuracy (79.86%) that might be insufficient depending on the type of application to be controlled. On the other hand, C016 excelled in terms of comfort, being significantly more comfortable than C001. Additionally, C016 achieved a remarkable mean accuracy of 96.53% and 164.54 bits/min with a trial duration of 1.05s. However, it is worth noting that starting from 4 cycles onwards (2.1 s), all conditions achieved an accuracy above 95%. Therefore, the choice of spatial frequency will depend on the application's characteristics and user requirements (e.g., the need for high accuracy, selection time, or session duration).

BCIs based on c-VEPs are innovative systems that have shown promising performance, but there is still much to explore regarding the visual parameters that influence system usability. Therefore, the findings obtained in this study can provide valuable insights for the design of upcoming c-VEP-based BCIs. Additionally, our results concerning stimulus spatial frequency have raised new research questions for future investigations. In conclusion, this study has demonstrated that stimulus spatial frequency has a significant impact on the performance of the c-VEP-based BCI, the comfort experienced by users during control, and their brain responses. These findings have important implications for the visual design that should be considered in future proposals for c-VEP-based BCIs.

## Data availability statement

The raw data supporting the conclusions of this article will be made available by the authors, without undue reservation.

## Ethics statement

The studies involving humans were approved by Research Ethics Committee, University of Valladolid. The studies were conducted in accordance with the local legislation and institutional requirements. The participants provided their written informed consent to participate in this study.

## Author contributions

ÁF-R: Conceptualization, Formal analysis, Investigation, Methodology, Validation, Visualization, Writing—original draft, Writing—review & editing. VM-C: Conceptualization, Data curation, Formal analysis, Methodology, Software, Validation, Writing—original draft, Writing—review & editing. ES-V: Writing—review & editing. RR-A: Writing—review & editing. RH: Funding acquisition, Project administration, Resources, Supervision, Writing—review & editing.
