# Supplementary material for: Influence of spatial frequency in visual stimuli for cVEP-based BCIs: evaluation of performance and user experience
Source: Front Hum Neurosci. 2023 Nov 10;17:1288438. doi: 10.3389/fnhum.2023.1288438 (PMC10667696; doi:10.3389/fnhum.2023.1288438)
Supplement: Supplementary file 1 [file Data_Sheet_1.pdf]

Supplementary Material

1 LAYOUT OPTIMIZATION

A standard GA was proposed to mitigate cross-talk between adjacent temporal shifts within the commands' encoding. Given an m-sequence of length L, m commands to encode (matrix dimensions of $N_{row} \times N_{col}$), and a lag array $\boldsymbol{l}$ (e.g., $[0, \tau, 2\tau, \ldots, (m-1)\tau]$ for equidistant lags with step τ), the objective of the algorithm is to maximize the distance between consecutive pair-wise lags across the entire m-sequence. First, a population of N_{chr} chromosomes of $N_{row} \times N_{col}$ dimensions is initialized by randomly shuffling and reshaping the $\boldsymbol{l}$ array n_{chr} times. Each chromosome represents a possible solution by assigning a lag for each cell command. The structure of the loop is standard, including: (1) fitness evaluation, (2) child selection, (3) cross-over, (4) mutation, and (5) elitism.

The pseudo-code is as follows:

1. Initialize the $\boldsymbol{X}$ population of dimensions $N_{chr} \times N_{row} \times N_{col}$ by shuffling the $\boldsymbol{l}$ array.
2. Set generation $= 0$.
3. While generation $<$ max_gen:
   3.1. Increment generation $+= 1$.
   3.2. Compute the fitness of each chromosome by summing the consecutive lag distances within the horizontal, vertical, and diagonal planes of each cell. It should be noted that the distance measurement needs to account for the cyclic nature of the m-sequence, as it repeats itself after L samples. Consequently, the distance is determined by $d(a,b) = \min(|a,b|, L-|a-b|)$. For a given chromosome $\boldsymbol{X}^{(n)} \in \mathbb{R}^{N_{row},N_{col}}$, the fitness can be calculated using the following equations:

$$\text{fitness}^{(n)} = \sum_{i=0}^{N_{row}} \sum_{\substack{j=0 \\ j \neq i}}^{N_{col}} d_{hor}(\boldsymbol{X}_{i,j}^{(n)}) + d_{ver}(\boldsymbol{X}_{i,j}^{(n)}) + d_{diag}(\boldsymbol{X}_{i,j}^{(n)}), \tag{S1}$$

$$d_{hor}(\boldsymbol{X}_{i,j}^{(n)}) = d(\boldsymbol{X}_{i,j}^{(n)}, \boldsymbol{X}_{i,j-1}^{(n)}) + d(\boldsymbol{X}_{i,j}^{(n)}, \boldsymbol{X}_{i,j+1}^{(n)}), \tag{S2}$$

$$d_{ver}(\boldsymbol{X}_{i,j}^{(n)}) = d(\boldsymbol{X}_{i,j}^{(n)}, \boldsymbol{X}_{i-1,j}^{(n)}) + d(\boldsymbol{X}_{i,j}^{(n)}, \boldsymbol{X}_{i+1,j}^{(n)}), \tag{S3}$$

$$\begin{aligned} d_{diag}(\boldsymbol{X}_{i,j}^{(n)}) =& d(\boldsymbol{X}_{i,j}^{(n)}, \boldsymbol{X}_{i-1,j-1}^{(n)}) + d(\boldsymbol{X}_{i,j}^{(n)}, \boldsymbol{X}_{i+1,j+1}^{(n)}) + \\ & d(\boldsymbol{X}_{i,j}^{(n)}, \boldsymbol{X}_{i-1,j+1}^{(n)}) + d(\boldsymbol{X}_{i,j}^{(n)}, \boldsymbol{X}_{i+1,j-1}^{(n)}) \end{aligned} \tag{S4}$$

   It is important to highlight that the distances d_{hor}, d_{ver}, and d_{diag} exclusively take into account the positions that are contained within the chromosome matrix. Consequently, any non-existent cells are disregarded in the distance calculations.
   3.3. If the fitness associated with a chromosome surpasses the current maximum fitness value, denoted as f_{max}, the maximum fitness is updated, and the chromosome is stored as the best solution identified thus far, represented as $\boldsymbol{X}_{best}$.
   3.4. Generate two child populations ($\boldsymbol{P}_1$ and $\boldsymbol{P}_2$) of $\lfloor N_{chr}/2 \rceil$ chromosomes each via k-tournament selection. In this approach, for each child chromosome, k parents are randomly selected from the

Table S1. Genetic algorithm hyperparameters for layout optimization

Parameter	Value	Description
N_{chr}	50	No. chromosomes
max_gen	1000	No. generations
k	2	*Size of tournament selection
p_c	0.9	*Probability of cross-over
p_m	$1/m$	*Probability of mutation

*Based on the recommended values from Deb et al. (2002) (2).

parent population $\boldsymbol{X}$, and their fitness values are compared. The parent with the highest fitness value is then chosen as the child chromosome.

3.5. Apply order cross-over (OX) to $\boldsymbol{P}_1$ and $\boldsymbol{P}_2$, as described in Davis (1985) (1). For the first child, extract a substring from a parent selected from $\boldsymbol{P}_1$ and produce a proto-child by copying it in into the corresponding position. Next, the second parent from $\boldsymbol{P}_2$ is chosen, and the lags not present in the proto-child are identified. These remaining lags are then inserted in order into the unfixed positions of the proto-child. The same procedure is repeated for the second child, utilizing the other parent. Finally, the resulting children are combined to form a new population $\boldsymbol{P}_3 \in \mathbb{R}^{N_{chr},N_{row},N_{col}}$. For each couple, OX is applied with a probability of p_c.

3.6. Apply a single-couple swap mutation operation with a probability of p_m. For every chromosome within $\boldsymbol{P}_3$, the algorithm randomly selects a pair of lags and exchanges their positions within the chromosome matrix.

3.7. Apply elitism to ensure that the highest-performing chromosome remains in the population for the subsequent generation. To maintain consistent dimensions, one chromosome is randomly removed from $\boldsymbol{P}_3$, resulting in $\hat{\boldsymbol{P}}_3 \in \mathbb{R}^{N_{chr}-1,N_{row},N_{col}}$. Subsequently, the population is updated as follows: $\boldsymbol{X}[\hat{\boldsymbol{P}}_3, \boldsymbol{X}_{best}]$.

4. Return the arrangement $\boldsymbol{X}_{best}$.

The chosen hyperparameters are shown in table S1. The optimization of large layouts often necessitates multiple iterations of the GA. In contrast, the optimization process for our 3×3 layout typically achieves convergence prior to reaching the designated maximum number of generations, i.e. max_gen. Notably, we have successfully enhanced the command arrangement from a fitness value of 420 (corresponding to the adjacent-lags layout) to 546 (optimal arrangement, shown in figure 3(D) of the original manuscript).

REFERENCES

[1]Davis, L. (1985, August). Applying adaptive algorithms to epistatic domains. In *IJCAI* (Vol. 85, pp. 162-164).

[2]Deb, K., Pratap, A., Agarwal, S., Meyarivan, T. A. M. T. (2002). A fast and elitist multiobjective genetic algorithm: NSGA-II. *IEEE transactions on evolutionary computation*, 6(2), 182-197.

2 SUPPLEMENTARY DATA

2.1 Individual averaged brain responses for each participant and condition

As stated in the manuscript, the averaged visual evoked potentials (VEPs) of calibration epochs for all conditions are depicted in the figure S1. Note that the VEPs were extracted from the Oz location. In order to extract them, the signals underwent pre-processing, which involved the application of two series of 7th-order infinite impulse response (IIR) Butterworth filters: (1) bandpass filter between 1-60 Hz, and (2) notch filter between 49-51 Hz to eliminate power line interference.

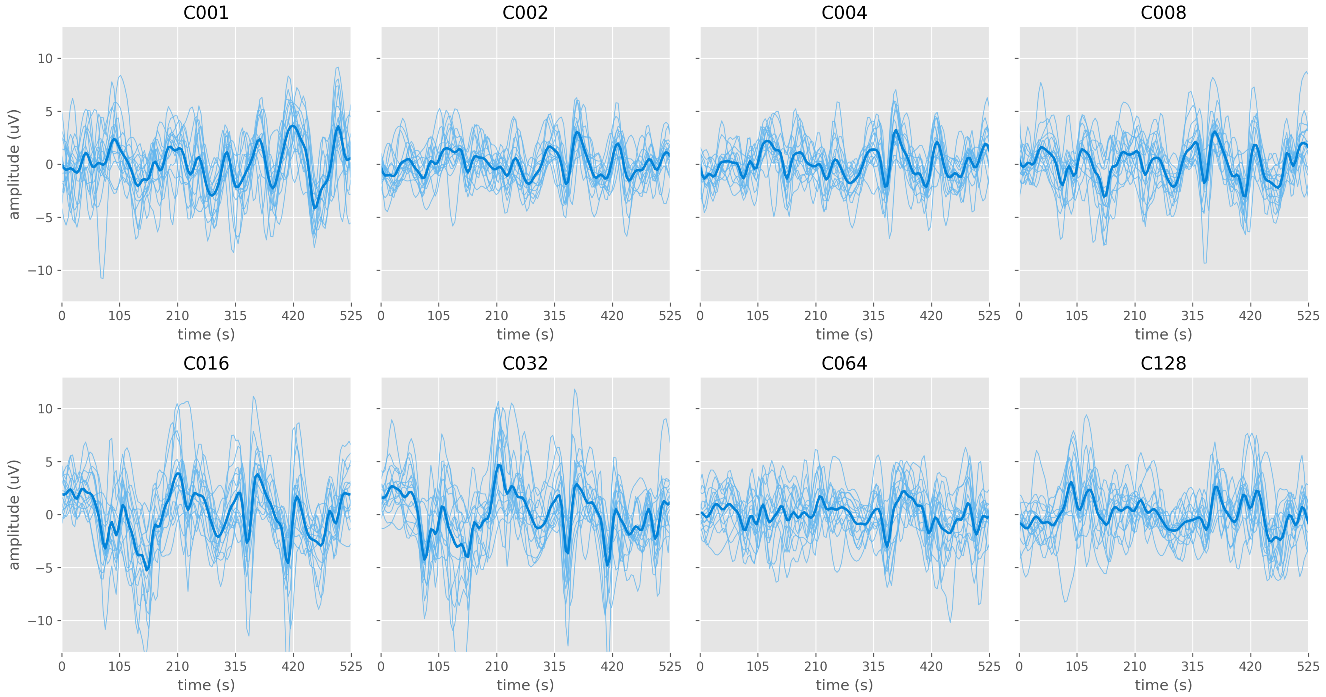

Figure S1. Individual visual evoked potentials (VEPs) for all conditions over Oz. Individual lines represent the averaged VEPs of the calibration epochs for each participant, whereas thick lines indicate the grand-averaged VEP among participants.

2.2 Unfolded performance results for each participant and condition

As stated in the manuscript, unfolded online results for each participant are available in the following tables S1-S8, indicated the reached accuracy, information transfer rate (ITR) and trial duration for each condition (C001, C002, C004, C008, C016, C032, C064, C128).

Table S2. Online performance results for condition C001

	User	No. cycles 1	2	3	4	5	6	7	8
Accuracy (%)	SF01	100.00	100.00	100.00	100.00	100.00	100.00	100.00	100.00
	SF02	88.89	94.44	100.00	100.00	100.00	100.00	100.00	100.00
	SF03	83.33	100.00	100.00	100.00	100.00	100.00	100.00	100.00
	SF04	94.44	94.44	100.00	100.00	100.00	100.00	100.00	100.00
	SF05	77.78	88.89	94.44	100.00	100.00	100.00	100.00	100.00
	SF06	88.89	100.00	100.00	100.00	100.00	100.00	100.00	100.00
	SF07	88.89	100.00	100.00	100.00	100.00	100.00	100.00	100.00
	SF08	94.44	100.00	94.44	100.00	100.00	100.00	100.00	100.00
	SF09	88.89	100.00	100.00	100.00	100.00	100.00	100.00	100.00
	SF10	55.56	88.89	83.33	94.44	100.00	100.00	100.00	100.00
	SF11	83.33	94.44	100.00	100.00	100.00	100.00	100.00	100.00
	SF12	72.22	83.33	83.33	83.33	83.33	83.33	83.33	83.33
	SF13	61.11	77.78	72.22	72.22	77.78	94.44	100.00	100.00
	SF14	55.56	94.44	100.00	100.00	100.00	100.00	100.00	100.00
	SF15	83.33	100.00	100.00	100.00	100.00	100.00	100.00	100.00
	SF16	61.11	94.44	94.44	100.00	100.00	100.00	100.00	100.00
	**Mean**	**79.86**	**94.44**	**95.14**	**96.87**	**97.57**	**98.61**	**98.96**	**98.96**
	STD	14.01	6.51	8.07	7.60	6.51	4.17	4.03	4.03
ITR (bits/min)	SF01	362.28	181.14	120.76	90.57	72.46	60.38	51.75	45.28
	SF02	266.67	153.93	120.76	90.57	72.46	60.38	51.75	45.28
	SF03	230.85	181.14	120.76	90.57	72.46	60.38	51.75	45.28
	SF04	307.85	153.93	120.76	90.57	72.46	60.38	51.75	45.28
	SF05	198.75	133.33	102.62	90.57	72.46	60.38	51.75	45.28
	SF06	266.67	181.14	120.76	90.57	72.46	60.38	51.75	45.28
	SF07	266.67	181.14	120.76	90.57	72.46	60.38	51.75	45.28
	SF08	307.85	181.14	102.62	90.57	72.46	60.38	51.75	45.28
	SF09	266.67	181.14	120.76	90.57	72.46	60.38	51.75	45.28
	SF10	96.63	133.33	76.95	76.96	72.46	60.38	51.75	45.28
	SF11	230.85	153.93	120.76	90.57	72.46	60.38	51.75	45.28
	SF12	169.62	115.42	76.95	57.71	46.17	38.47	32.98	28.86
	SF13	118.76	99.37	56.54	42.41	39.75	51.31	51.75	45.28
	SF14	96.63	153.93	120.76	90.57	72.46	60.38	51.75	45.28
	SF15	230.85	181.14	120.76	90.57	72.46	60.38	51.75	45.28
	SF16	118.76	153.93	102.62	90.57	72.46	60.38	51.75	45.28
	**Mean**	**221.02**	**157.44**	**107.87**	**84.66**	**68.77**	**58.44**	**50.58**	**44.26**
	STD	78.52	25.36	19.83	13.75	9.82	5.60	4.54	3.98
**Duration (s)**		0.525	1.050	1.575	2.100	2.625	3.150	3.675	4.200

Table S3. Online performance results for condition C002

	User	No. cycles 1	2	3	4	5	6	7	8
Accuracy (%)	SF01	83.33	100.00	100.00	100.00	100.00	100.00	100.00	100.00
	SF02	55.56	94.44	100.00	100.00	100.00	100.00	100.00	100.00
	SF03	55.56	77.78	94.44	100.00	100.00	100.00	100.00	100.00
	SF04	83.33	100.00	100.00	100.00	100.00	100.00	100.00	100.00
	SF05	72.22	94.44	88.89	88.89	100.00	100.00	100.00	100.00
	SF06	55.56	77.78	83.33	88.89	94.44	94.44	94.44	100.00
	SF07	55.56	83.33	83.33	94.44	100.00	100.00	100.00	100.00
	SF08	88.89	94.44	94.44	94.44	94.44	100.00	100.00	100.00
	SF09	66.67	88.89	94.44	94.44	94.44	100.00	100.00	100.00
	SF10	44.44	83.33	88.89	100.00	100.00	100.00	100.00	100.00
	SF11	50.00	72.22	94.44	100.00	100.00	100.00	100.00	100.00
	SF12	77.78	100.00	100.00	100.00	100.00	100.00	100.00	100.00
	SF13	44.44	72.22	83.33	83.33	83.33	83.33	83.33	83.33
	SF14	55.56	83.33	94.44	100.00	100.00	94.44	100.00	100.00
	SF15	61.11	100.00	100.00	100.00	100.00	100.00	100.00	100.00
	SF16	50.00	94.44	88.89	100.00	100.00	100.00	100.00	100.00
	**Mean**	**62.50**	**88.54**	**93.06**	**96.53**	**97.92**	**98.26**	**98.61**	**98.96**
	STD	13.96	9.72	6.05	5.15	4.34	4.27	4.17	4.03
ITR (bits/min)	SF01	230.85	181.14	120.76	90.57	72.46	60.38	51.75	45.28
	SF02	96.63	153.93	120.76	90.57	72.46	60.38	51.75	45.28
	SF03	96.63	99.37	102.62	90.57	72.46	60.38	51.75	45.28
	SF04	230.85	181.14	120.76	90.57	72.46	60.38	51.75	45.28
	SF05	169.62	153.93	88.89	66.67	72.46	60.38	51.75	45.28
	SF06	96.63	99.37	76.95	66.67	61.57	51.31	43.98	45.28
	SF07	96.63	115.42	76.95	76.96	72.46	60.38	51.75	45.28
	SF08	266.67	153.93	102.62	76.96	61.57	60.38	51.75	45.28
	SF09	143.04	133.33	102.62	76.96	61.57	60.38	51.75	45.28
	SF10	58.54	115.42	88.89	90.57	72.46	60.38	51.75	45.28
	SF11	76.56	84.81	102.62	90.57	72.46	60.38	51.75	45.28
	SF12	198.75	181.14	120.76	90.57	72.46	60.38	51.75	45.28
	SF13	58.54	84.81	76.95	57.71	46.17	38.47	32.98	28.86
	SF14	96.63	115.42	102.62	90.57	72.46	51.31	51.75	45.28
	SF15	118.76	181.14	120.76	90.57	72.46	60.38	51.75	45.28
	SF16	76.56	153.93	88.89	90.57	72.46	60.38	51.75	45.28
	**Mean**	**131.99**	**136.76**	**100.90**	**82.98**	**68.77**	**57.88**	**50.09**	**44.26**
	STD	64.73	34.12	16.09	10.77	7.20	5.83	4.80	3.98
**Duration (s)**		0.525	1.050	1.575	2.100	2.625	3.150	3.675	4.200

Table S4. Online performance results for condition C004

		No. cycles							
	**User**	**1**	**2**	**3**	**4**	**5**	**6**	**7**	**8**
**Accuracy (%)**	SF01	77.78	100.00	100.00	100.00	100.00	100.00	100.00	100.00
	SF02	50.00	100.00	100.00	100.00	100.00	100.00	100.00	100.00
	SF03	44.44	94.44	100.00	100.00	100.00	100.00	100.00	100.00
	SF04	83.33	100.00	100.00	100.00	100.00	100.00	100.00	100.00
	SF05	88.89	100.00	100.00	100.00	100.00	100.00	100.00	100.00
	SF06	66.67	94.44	100.00	100.00	100.00	94.44	100.00	100.00
	SF07	38.89	72.22	88.89	88.89	94.44	94.44	100.00	100.00
	SF08	88.89	94.44	100.00	100.00	100.00	100.00	100.00	100.00
	SF09	61.11	94.44	100.00	100.00	100.00	100.00	100.00	100.00
	SF10	61.11	83.33	94.44	100.00	100.00	100.00	100.00	100.00
	SF11	61.11	88.89	100.00	100.00	100.00	100.00	100.00	100.00
	SF12	77.78	94.44	94.44	94.44	94.44	94.44	94.44	94.44
	SF13	66.67	94.44	100.00	100.00	100.00	100.00	100.00	100.00
	SF14	33.33	66.67	77.78	83.33	88.89	88.89	88.89	88.89
	SF15	83.33	100.00	100.00	100.00	100.00	100.00	100.00	100.00
	SF16	44.44	83.33	100.00	100.00	100.00	100.00	100.00	100.00
	**Mean**	**64.24**	**91.32**	**97.22**	**97.92**	**98.61**	**98.26**	**98.96**	**98.96**
	STD	17.56	9.81	5.89	4.76	3.11	3.24	2.93	2.93
**ITR (bits/min)**	SF01	198.75	181.14	120.76	90.57	72.46	60.38	51.75	45.28
	SF02	76.56	181.14	120.76	90.57	72.46	60.38	51.75	45.28
	SF03	58.54	153.93	120.76	90.57	72.46	60.38	51.75	45.28
	SF04	230.85	181.14	120.76	90.57	72.46	60.38	51.75	45.28
	SF05	266.67	181.14	120.76	90.57	72.46	60.38	51.75	45.28
	SF06	143.04	153.93	120.76	90.57	72.46	51.31	51.75	45.28
	SF07	42.57	84.81	88.89	66.67	61.57	51.31	51.75	45.28
	SF08	266.67	153.93	120.76	90.57	72.46	60.38	51.75	45.28
	SF09	118.76	153.93	120.76	90.57	72.46	60.38	51.75	45.28
	SF10	118.76	115.42	102.62	90.57	72.46	60.38	51.75	45.28
	SF11	118.76	133.33	120.76	90.57	72.46	60.38	51.75	45.28
	SF12	198.75	153.93	102.62	76.96	61.57	51.31	43.98	38.48
	SF13	143.04	153.93	120.76	90.57	72.46	60.38	51.75	45.28
	SF14	28.76	71.52	66.25	57.71	53.33	44.44	38.10	33.33
	SF15	230.85	181.14	120.76	90.57	72.46	60.38	51.75	45.28
	SF16	58.54	115.42	120.76	90.57	72.46	60.38	51.75	45.28
	**Mean**	**143.74**	**146.86**	**113.09**	**86.17**	**69.90**	**57.68**	**50.41**	**44.11**
	STD	77.38	33.41	15.22	9.77	5.58	4.90	3.69	3.23
**Duration (s)**		0.525	1.050	1.575	2.100	2.625	3.150	3.675	4.200

Table S5. Online performance results for condition C008

	User	No. cycles 1	2	3	4	5	6	7	8
Accuracy (%)	SF01	77.78	100.00	100.00	100.00	100.00	100.00	100.00	100.00
	SF02	61.11	88.89	94.44	100.00	100.00	100.00	100.00	100.00
	SF03	66.67	100.00	100.00	100.00	100.00	100.00	100.00	100.00
	SF04	94.44	100.00	100.00	100.00	100.00	100.00	100.00	100.00
	SF05	72.22	94.44	94.44	100.00	100.00	100.00	100.00	100.00
	SF06	66.67	83.33	94.44	100.00	100.00	100.00	100.00	100.00
	SF07	44.44	88.89	88.89	94.44	94.44	100.00	100.00	100.00
	SF08	55.56	94.44	100.00	100.00	100.00	100.00	100.00	100.00
	SF09	83.33	100.00	100.00	100.00	100.00	100.00	100.00	100.00
	SF10	72.22	94.44	100.00	100.00	100.00	100.00	100.00	100.00
	SF11	72.22	88.89	94.44	94.44	100.00	100.00	100.00	100.00
	SF12	88.89	94.44	100.00	100.00	100.00	100.00	100.00	100.00
	SF13	33.33	72.22	83.33	94.44	100.00	100.00	100.00	100.00
	SF14	44.44	77.78	83.33	88.89	100.00	100.00	100.00	100.00
	SF15	83.33	100.00	100.00	100.00	100.00	100.00	100.00	100.00
	SF16	66.67	77.78	88.89	94.44	94.44	100.00	100.00	100.00
	**Mean**	**67.71**	**90.97**	**95.14**	**97.92**	**99.31**	**100.00**	**100.00**	**100.00**
	STD	16.34	8.76	5.85	3.33	1.84	0.00	0.00	0.00
ITR (bits/min)	SF01	198.75	181.14	120.76	90.57	72.46	60.38	51.75	45.28
	SF02	118.76	133.33	102.62	90.57	72.46	60.38	51.75	45.28
	SF03	143.04	181.14	120.76	90.57	72.46	60.38	51.75	45.28
	SF04	307.85	181.14	120.76	90.57	72.46	60.38	51.75	45.28
	SF05	169.62	153.93	102.62	90.57	72.46	60.38	51.75	45.28
	SF06	143.04	115.42	102.62	90.57	72.46	60.38	51.75	45.28
	SF07	58.54	133.33	88.89	76.96	61.57	60.38	51.75	45.28
	SF08	96.63	153.93	120.76	90.57	72.46	60.38	51.75	45.28
	SF09	230.85	181.14	120.76	90.57	72.46	60.38	51.75	45.28
	SF10	169.62	153.93	120.76	90.57	72.46	60.38	51.75	45.28
	SF11	169.62	133.33	102.62	76.96	72.46	60.38	51.75	45.28
	SF12	266.67	153.93	120.76	90.57	72.46	60.38	51.75	45.28
	SF13	28.76	84.81	76.95	76.96	72.46	60.38	51.75	45.28
	SF14	58.54	99.37	76.95	66.67	72.46	60.38	51.75	45.28
	SF15	230.85	181.14	120.76	90.57	72.46	60.38	51.75	45.28
	SF16	143.04	99.37	88.89	76.96	61.57	60.38	51.75	45.28
	**Mean**	**158.39**	**145.02**	**106.76**	**85.67**	**71.09**	**60.38**	**51.75**	**45.28**
	STD	74.75	31.54	15.92	7.62	3.60	0.00	0.00	0.00
**Duration (s)**		0.525	1.050	1.575	2.100	2.625	3.150	3.675	4.200

Table S6. Online performance results for condition C016

	User	No. cycles 1	2	3	4	5	6	7	8
Accuracy (%)	SF01	61.11	94.44	100.00	100.00	100.00	100.00	100.00	100.00
	SF02	55.56	94.44	100.00	100.00	100.00	100.00	100.00	100.00
	SF03	66.67	100.00	100.00	100.00	100.00	100.00	100.00	100.00
	SF04	100.00	100.00	100.00	100.00	100.00	100.00	100.00	100.00
	SF05	77.78	94.44	100.00	100.00	100.00	100.00	100.00	100.00
	SF06	72.22	94.44	94.44	94.44	94.44	94.44	94.44	94.44
	SF07	61.11	100.00	100.00	100.00	100.00	100.00	100.00	100.00
	SF08	50.00	88.89	94.44	94.44	100.00	100.00	100.00	100.00
	SF09	94.44	100.00	100.00	100.00	100.00	100.00	100.00	100.00
	SF10	55.56	94.44	100.00	100.00	100.00	100.00	100.00	100.00
	SF11	61.11	94.44	100.00	100.00	100.00	100.00	100.00	100.00
	SF12	72.22	100.00	100.00	100.00	100.00	100.00	100.00	100.00
	SF13	83.33	100.00	100.00	100.00	100.00	100.00	100.00	100.00
	SF14	38.89	94.44	100.00	100.00	100.00	100.00	100.00	100.00
	SF15	72.22	100.00	100.00	100.00	100.00	100.00	100.00	100.00
	SF16	44.44	94.44	100.00	100.00	100.00	100.00	100.00	100.00
	**Mean**	**66.67**	**96.53**	**99.31**	**99.31**	**99.65**	**99.65**	**99.65**	**99.65**
	STD	16.32	3.33	1.84	1.84	1.34	1.34	1.34	1.34
ITR (bits/min)	SF01	118.76	153.93	120.76	90.57	72.46	60.38	51.75	45.28
	SF02	96.63	153.93	120.76	90.57	72.46	60.38	51.75	45.28
	SF03	143.04	181.14	120.76	90.57	72.46	60.38	51.75	45.28
	SF04	362.28	181.14	120.76	90.57	72.46	60.38	51.75	45.28
	SF05	198.75	153.93	120.76	90.57	72.46	60.38	51.75	45.28
	SF06	169.62	153.93	102.62	76.96	61.57	51.31	43.98	38.48
	SF07	118.76	181.14	120.76	90.57	72.46	60.38	51.75	45.28
	SF08	76.56	133.33	102.62	76.96	72.46	60.38	51.75	45.28
	SF09	307.85	181.14	120.76	90.57	72.46	60.38	51.75	45.28
	SF10	96.63	153.93	120.76	90.57	72.46	60.38	51.75	45.28
	SF11	118.76	153.93	120.76	90.57	72.46	60.38	51.75	45.28
	SF12	169.62	181.14	120.76	90.57	72.46	60.38	51.75	45.28
	SF13	230.85	181.14	120.76	90.57	72.46	60.38	51.75	45.28
	SF14	42.57	153.93	120.76	90.57	72.46	60.38	51.75	45.28
	SF15	169.62	181.14	120.76	90.57	72.46	60.38	51.75	45.28
	SF16	58.54	153.93	120.76	90.57	72.46	60.38	51.75	45.28
	**Mean**	**154.93**	**164.54**	**118.49**	**88.87**	**71.78**	**59.81**	**51.27**	**44.86**
	STD	84.27	15.42	6.00	4.50	2.63	2.20	1.88	1.65
**Duration (s)**		0.525	1.050	1.575	2.100	2.625	3.150	3.675	4.200

Table S7. Online performance results for condition C032

	User	No. cycles 1	2	3	4	5	6	7	8
Accuracy (%)	SF01	83.33	94.44	94.44	94.44	94.44	94.44	94.44	94.44
	SF02	72.22	100.00	100.00	100.00	100.00	100.00	100.00	100.00
	SF03	83.33	100.00	100.00	100.00	100.00	100.00	100.00	100.00
	SF04	88.89	100.00	100.00	100.00	100.00	100.00	100.00	100.00
	SF05	83.33	100.00	100.00	100.00	100.00	100.00	100.00	100.00
	SF06	66.67	83.33	88.89	88.89	88.89	94.44	94.44	94.44
	SF07	38.89	94.44	100.00	100.00	100.00	100.00	100.00	100.00
	SF08	72.22	94.44	100.00	100.00	100.00	100.00	100.00	100.00
	SF09	88.89	100.00	100.00	100.00	100.00	100.00	100.00	100.00
	SF10	55.56	83.33	83.33	83.33	94.44	83.33	88.89	88.89
	SF11	50.00	100.00	100.00	100.00	100.00	100.00	100.00	100.00
	SF12	94.44	100.00	100.00	100.00	100.00	100.00	100.00	100.00
	SF13	72.22	100.00	100.00	100.00	100.00	100.00	100.00	100.00
	SF14	38.89	61.11	83.33	100.00	94.44	100.00	100.00	100.00
	SF15	61.11	88.89	100.00	100.00	100.00	100.00	100.00	100.00
	SF16	72.22	100.00	100.00	100.00	100.00	100.00	100.00	100.00
	**Mean**	**70.14**	**93.75**	**96.88**	**97.92**	**98.26**	**98.26**	**98.61**	**98.61**
	STD	16.77	10.18	5.88	4.76	3.24	4.27	3.11	3.11
ITR (bits/min)	SF01	230.85	153.93	102.62	76.96	61.57	51.31	43.98	38.48
	SF02	169.62	181.14	120.76	90.57	72.46	60.38	51.75	45.28
	SF03	230.85	181.14	120.76	90.57	72.46	60.38	51.75	45.28
	SF04	266.67	181.14	120.76	90.57	72.46	60.38	51.75	45.28
	SF05	230.85	181.14	120.76	90.57	72.46	60.38	51.75	45.28
	SF06	143.04	115.42	88.89	66.67	53.33	51.31	43.98	38.48
	SF07	42.57	153.93	120.76	90.57	72.46	60.38	51.75	45.28
	SF08	169.62	153.93	120.76	90.57	72.46	60.38	51.75	45.28
	SF09	266.67	181.14	120.76	90.57	72.46	60.38	51.75	45.28
	SF10	96.63	115.42	76.95	57.71	61.57	38.47	38.10	33.33
	SF11	76.56	181.14	120.76	90.57	72.46	60.38	51.75	45.28
	SF12	307.85	181.14	120.76	90.57	72.46	60.38	51.75	45.28
	SF13	169.62	181.14	120.76	90.57	72.46	60.38	51.75	45.28
	SF14	42.57	59.38	76.95	90.57	61.57	60.38	51.75	45.28
	SF15	118.76	133.33	120.76	90.57	72.46	60.38	51.75	45.28
	SF16	169.62	181.14	120.76	90.57	72.46	60.38	51.75	45.28
	**Mean**	**170.77**	**157.22**	**112.16**	**86.17**	**69.22**	**57.88**	**49.93**	**43.69**
	STD	78.55	34.29	15.81	9.77	5.88	5.83	3.99	3.49
**Duration (s)**		0.525	1.050	1.575	2.100	2.625	3.150	3.675	4.200

Table S8. Online performance results for condition C064

	User	No. cycles 1	2	3	4	5	6	7	8
Accuracy (%)	SF01	61.11	100.00	100.00	100.00	100.00	100.00	100.00	100.00
	SF02	61.11	100.00	100.00	100.00	100.00	100.00	100.00	100.00
	SF03	66.67	100.00	100.00	100.00	100.00	100.00	100.00	100.00
	SF04	50.00	100.00	100.00	100.00	100.00	100.00	100.00	100.00
	SF05	44.44	66.67	83.33	88.89	88.89	88.89	94.44	94.44
	SF06	50.00	77.78	83.33	88.89	83.33	83.33	94.44	88.89
	SF07	61.11	88.89	88.89	94.44	100.00	94.44	100.00	100.00
	SF08	66.67	83.33	100.00	100.00	100.00	100.00	100.00	100.00
	SF09	77.78	100.00	100.00	100.00	100.00	100.00	100.00	100.00
	SF10	27.78	61.11	88.89	100.00	100.00	100.00	100.00	100.00
	SF11	61.11	94.44	100.00	100.00	100.00	100.00	100.00	100.00
	SF12	66.67	77.78	94.44	88.89	100.00	100.00	100.00	100.00
	SF13	55.56	72.22	94.44	100.00	100.00	100.00	100.00	100.00
	SF14	55.56	66.67	77.78	94.44	94.44	94.44	94.44	100.00
	SF15	61.11	88.89	100.00	100.00	100.00	100.00	100.00	100.00
	SF16	55.56	72.22	77.78	77.78	83.33	83.33	88.89	83.33
	**Mean**	**57.64**	**84.38**	**93.06**	**95.83**	**96.87**	**96.53**	**98.26**	**97.92**
	STD	10.91	13.50	8.22	6.36	5.88	5.85	3.24	4.76
ITR (bits/min)	SF01	118.76	181.14	120.76	90.57	72.46	60.38	51.75	45.28
	SF02	118.76	181.14	120.76	90.57	72.46	60.38	51.75	45.28
	SF03	143.04	181.14	120.76	90.57	72.46	60.38	51.75	45.28
	SF04	76.56	181.14	120.76	90.57	72.46	60.38	51.75	45.28
	SF05	58.54	71.52	76.95	66.67	53.33	44.44	43.98	38.48
	SF06	76.56	99.37	76.95	66.67	46.17	38.47	43.98	33.33
	SF07	118.76	133.33	88.89	76.96	72.46	51.31	51.75	45.28
	SF08	143.04	115.42	120.76	90.57	72.46	60.38	51.75	45.28
	SF09	198.75	181.14	120.76	90.57	72.46	60.38	51.75	45.28
	SF10	17.24	59.38	88.89	90.57	72.46	60.38	51.75	45.28
	SF11	118.76	153.93	120.76	90.57	72.46	60.38	51.75	45.28
	SF12	143.04	99.37	102.62	66.67	72.46	60.38	51.75	45.28
	SF13	96.63	84.81	102.62	90.57	72.46	60.38	51.75	45.28
	SF14	96.63	71.52	66.25	76.96	61.57	51.31	43.98	45.28
	SF15	118.76	133.33	120.76	90.57	72.46	60.38	51.75	45.28
	SF16	96.63	84.81	66.25	49.69	46.17	38.47	38.10	28.86
	**Mean**	**108.78**	**125.78**	**102.22**	**81.83**	**67.29**	**55.51**	**49.44**	**43.09**
	STD	39.95	44.29	20.87	12.58	9.49	7.90	4.20	4.88
**Duration (s)**		0.525	1.050	1.575	2.100	2.625	3.150	3.675	4.200

Table S9. Online performance results for condition C128

	User	No. cycles 1	2	3	4	5	6	7	8
Accuracy (%)	SF01	55.56	66.67	77.78	88.89	94.44	94.44	94.44	94.44
	SF02	61.11	94.44	100.00	100.00	100.00	100.00	100.00	100.00
	SF03	61.11	88.89	94.44	100.00	100.00	100.00	100.00	100.00
	SF04	88.89	94.44	100.00	100.00	100.00	100.00	100.00	100.00
	SF05	44.44	44.44	66.67	77.78	77.78	83.33	83.33	83.33
	SF06	77.78	83.33	100.00	94.44	100.00	100.00	100.00	100.00
	SF07	61.11	94.44	100.00	100.00	100.00	100.00	100.00	100.00
	SF08	88.89	100.00	100.00	100.00	100.00	100.00	100.00	100.00
	SF09	61.11	88.89	94.44	100.00	100.00	100.00	100.00	100.00
	SF10	72.22	100.00	100.00	100.00	100.00	100.00	100.00	100.00
	SF11	72.22	94.44	94.44	100.00	100.00	100.00	100.00	100.00
	SF12	72.22	100.00	94.44	100.00	100.00	100.00	100.00	100.00
	SF13	61.11	83.33	88.89	88.89	88.89	88.89	94.44	94.44
	SF14	50.00	77.78	88.89	100.00	100.00	100.00	100.00	100.00
	SF15	61.11	66.67	77.78	88.89	94.44	88.89	88.89	88.89
	SF16	55.56	66.67	83.33	88.89	83.33	83.33	83.33	83.33
	**Mean**	**65.28**	**84.03**	**91.32**	**95.49**	**96.18**	**96.18**	**96.53**	**96.53**
	STD	12.19	15.33	9.81	6.58	6.72	6.12	5.85	5.85
ITR (bits/min)	SF01	96.63	71.52	66.25	66.67	61.57	51.31	43.98	38.48
	SF02	118.76	153.93	120.76	90.57	72.46	60.38	51.75	45.28
	SF03	118.76	133.33	102.62	90.57	72.46	60.38	51.75	45.28
	SF04	266.67	153.93	120.76	90.57	72.46	60.38	51.75	45.28
	SF05	58.54	29.27	47.68	49.69	39.75	38.47	32.98	28.86
	SF06	198.75	115.42	120.76	76.96	72.46	60.38	51.75	45.28
	SF07	118.76	153.93	120.76	90.57	72.46	60.38	51.75	45.28
	SF08	266.67	181.14	120.76	90.57	72.46	60.38	51.75	45.28
	SF09	118.76	133.33	102.62	90.57	72.46	60.38	51.75	45.28
	SF10	169.62	181.14	120.76	90.57	72.46	60.38	51.75	45.28
	SF11	169.62	153.93	102.62	90.57	72.46	60.38	51.75	45.28
	SF12	169.62	181.14	102.62	90.57	72.46	60.38	51.75	45.28
	SF13	118.76	115.42	88.89	66.67	53.33	44.44	43.98	38.48
	SF14	76.56	99.37	88.89	90.57	72.46	60.38	51.75	45.28
	SF15	118.76	71.52	66.25	66.67	61.57	44.44	38.10	33.33
	SF16	96.63	71.52	76.95	66.67	46.17	38.47	32.98	28.86
	**Mean**	**142.62**	**124.99**	**98.12**	**81.19**	**66.21**	**55.08**	**47.58**	**41.63**
	STD	58.73	44.39	22.84	13.07	10.43	8.29	6.77	5.93
**Duration (s)**		0.525	1.050	1.575	2.100	2.625	3.150	3.675	4.200
